# Paeoniflorin Inhibits the Activation of Microglia and Alleviates Depressive Behavior by Regulating SIRT1-NF-kB-NLRP3/Pyroptosis Pathway

**DOI:** 10.3390/ijms252312543

**Published:** 2024-11-22

**Authors:** Xue Wang, Lili Su, Silu Liu, Zhongmei He, Jianming Li, Ying Zong, Weijia Chen, Rui Du

**Affiliations:** 1 College of Chinese Medicinal Materials, Jilin Agricultural University, Changchun 130118, China; 13889862812@163.com (X.W.); 13630524570@163.com (L.S.); liusilu0616@163.com (S.L.); heather78@126.com (Z.H.); m15568781138@163.com (J.L.); zongying7699@126.com (Y.Z.); 2 Jilin Provincial Engineering Research Center for Efficient Breeding and Product Development of Sika Deer, Changchun 130118, China; 3 Key Laboratory of Animal Production and Product Quality and Security, Ministry of Education, Ministry of National Education, Changchun 130118, China

**Keywords:** paeoniflorin, depression, NLRP3 inflammasome, pyroptosis, SIRT1

## Abstract

Inflammation assumes a vital role in the pathogenesis of depression and in antidepressant treatment. Paeoniflorin (PF), a monoterpene glycoside analog possessing anti-inflammatory attributes, exhibits therapeutic efficacy on depression-like behavior in mice. The objective of this study was to evaluate the antidepressant effects of PF on depression elicited by the chronic unpredictable mild stress (CUMS) model and the precise neural sequence associated with the inflammatory process. In this study, we established an in vivo mouse model induced by CUMS and an in vitro BV2 cell model induced by LPS+ATP. The mechanism of PF for depression was assessed by the SIRT1 selective inhibitor EX-527. The findings demonstrated that PF significantly alleviated the damage of BV2 cells treated with LPS and ATP, inhibited the generation of ROS, up-regulated the expression of SIRT1 mRNA, and down-regulated the expression of nuclear NF-κB, p65, NLRP3, Caspase-1 and GSDMD-N in vitro. In vivo, PF mitigated the depressive-like behavior induced by CUMS, reduced the number of neurons, and decreased the secretion of pro-inflammatory factors IL-1β, IL-6, and TNF-α in the hippocampus. Immunohistochemical results indicated that PF attenuated CUMS-induced hyperactivation of microglia. Moreover, the expression level of SIRT1 in the hippocampus was augmented, while the protein levels of NF-κB, p65, NLRP3, Caspase-1, IL-1β and GSDMD-N were diminished after PF treatment. Additionally, the selective inhibition of SIRT1 attenuated the therapeutic effect of PF on depression. These results imply that PF possesses antidepressant properties that rely on SIRT1 signaling to regulate NLRP3 inflammasome inactivation.

## 1. Introduction

Depression is one of the most common neuropsychiatric disorders, characterized by depression, lack of motivation and appetite, dysfunction of thinking, sleep and cognition, and even repeated suicidal thoughts [1]. According to the estimate of the World Health Organization, there are about 350 million people suffering from depression in the world, with a growth rate of about 18% in the past decade, and about 1 million people commit suicide due to depression every year [2]. A number of antidepressants are available for the treatment of depression, but they still have a number of limitations, including low cure rates and serious side effects [3]. Therefore, it is important to find more reliable methods to treat depression.

It is speculated that neuroinflammation, imbalance of neurotransmitters, excessive oxidative stress and imbalance of apoptosis all aggravate the development of depression [4]. In recent years, more and more studies have emphasized the relationship between depression, inflammation and pyroptosis. Neuroinflammation is considered to be an important and key component of the pathogenesis of neurodegenerative diseases and mental diseases [5]. Some scholars have found abnormal expressions of inflammatory factors and inflammation-related indicators in depression model animals [6], and we found that when the brain is stimulated by the outside world, it will induce neuroinflammation in the central nervous system, thus aggravating depression [7].

Pyroptosis is a pro-inflammatory form that regulates cell death [8]. It is characterized by the assembly and activation of NLRP3 inflammasome [9]. Once activated, NLRP3 inflammasome will aggregate and lead to various changes, including the recruitment of ASC and the self-activation of Caspase-1 into cleaved Caspase-1, which will promote the activation and cleavage of GSDMD to form GSDMD-N, induce pyroptosis and release pro-inflammatory factors [10]. Pyroptosis acts on the downstream of NLRP3 inflammasome, releasing IL-1β [11].

It is generally accepted that the transcription factor NF-κB drives the activation of NLRP3 inflammasome during pyroptosis [12]. Silent information regulatory factor related enzyme 1 (SIRT1) is an NAD^+^-dependent deacetylase with multiple functions in oxidative stress, inflammation and apoptosis [13]. SIRT1 signaling inactivates the deacetylated p65 subunit of NF-κB at the Lys310 site and ultimately inactivates the NLRP3 inflammasome [14]. The NLRP3 inflammasome is involved in the pathogenesis of depression and may serve as a novel therapeutic strategy [15].

Paeoniflorin is the main effective component of Radix Paeoniae Rubra and Radix Paeoniae Alba. A large number of studies have proven that paeoniflorin has anti-inflammatory, sedative and analgesic effects, anti-tumor, liver protection, neuroprotection and immunomodulation [16,17]. With the deepening of the research on paeoniflorin, more and more studies have found that paeoniflorin plays an important role in the treatment of depression [18]. It was found that the antidepressant activity of paeoniflorin may be due to the over-expression of brain-derived neurotrophic factor (BDNF) [19], the signal activation of ERK1/2 [20], the inhibition of signal transduction of TLR4/NF-κB/NLRP3 [21], the decrease of pro-inflammatory cytokines, the inhibition of pyroptosis of CASP-11-GSDND and the negative regulation of microglia activation [22]. However, the effects of paeoniflorin on depression and the SIRT1-NF-kB-NLRP3 signaling pathway have not been studied.

Therefore, this study aims to evaluate the pharmacological effect of paeoniflorin on depression and explore its possible mechanism. Firstly, in vitro, we studied the effect of paeoniflorin on inflammatory corpuscles and blepharoptosis of NLRP3 by using LPS and ATP-treated microglia BV2. Then, in vivo, we established a CUMS mice model to evaluate the effects of paeoniflorin on alleviating depression-like behavior and inhibiting the activation of inflammatory corpuscles and pyroptosis. Finally, the SIRT1 inhibitor EX-527 was used to verify the role of the SIRT1 signaling pathway in paeoniflorin-related antidepressant and anti-inflammatory activities.

## 2. Results

### 2.1. PF Inhibits the Activation of Indexes Related to Pyroptosis of BV2 Cells Induced by LPS and ATP and the Expression of Inflammatory Cytokines

**Figure 1:**
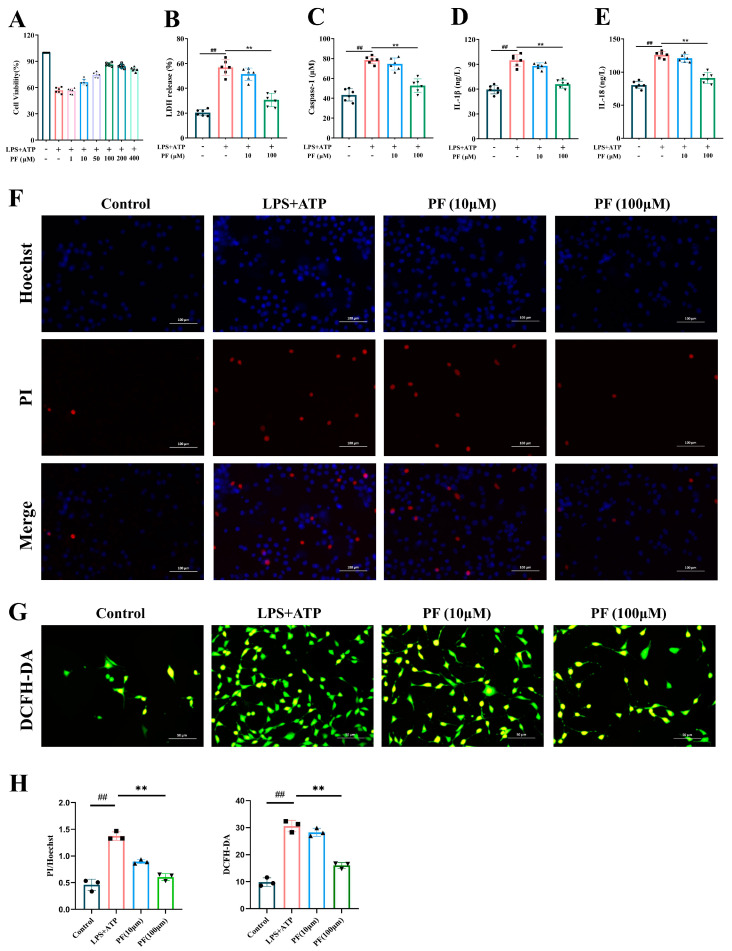
PF inhibited the activation of apoptosis, inflammation, pyroptosis and ROS of BV2 cells induced by LPS+ATP. (**A**) Cell viability of BV2. (**B**–**E**) The content of LDH, Caspase-1, IL-1β and IL-18. (**F**) Hoechst/PI double staining (×400). (**G**) ROS staining (×400). (**H**) Quantitative analysis of PI/Hoechst and DCFH-DA. Data are presented as mean ± SEM. ## *p* < 0.01, compared to control group; ** *p* < 0.01, compared to LPS+ATP group.

A CCK-8 kit was used to screen the best concentration of PF to BV2 cell model induced by LPS+ATP. After LPS+ATP induction, we found that the cell viability of BV2 increased in a dose-dependent manner and was optimal at 100 μM (Figure 1A).

Pyroptosis will lead to the formation of pore membranes, the release of lactate dehydrogenase (LDH), the activation of Caspase-1 and the secretion of inflammatory cytokines. The concentration of LDH, Caspase-1, IL-1β and IL-18 in the BV2 cell culture medium was detected to indirectly reflect the effect of PF on pyroptosis. It was found that LPS+ATP could induce the release of LDH (Figure 1B), the activation of caspase-1 (Figure 1C) and the secretion of IL-1β (Figure 1D) and IL-18 (Figure 1E) in BV2 cells. Conversely, the concentrations of LDH, Caspase-1, IL-1β and IL-18 decreased in a dose-dependent manner by PF treatment. It showed that PF inhibits the expression of inflammatory cytokines, which are related to pyroptosis.

### 2.2. PF Inhibited the Apoptosis Signal Enhancement and ROS Activation in Cultured BV2 Microglia Stimulated by LPS and ATP

Hoechst 33,258 could penetrate the cell membrane, enter normal cells and apoptotic cells to combine with DNA, and show blue fluorescence after staining, and the fluorescence intensity of apoptotic cells will be significantly enhanced than that of normal cells. However, PI could not stain normal cells or apoptotic cells with intact cell membranes. We found that the blue and red fluorescence of BV2 cells was enhanced after stimulation by LPS and ATP, which indicated that BV2 cells had apoptosis, while PF treatment reduced the number of apoptotic cells and inhibited the level of apoptosis (Figure 1F,H).

In order to explore the effect of PF on ROS activation in BV2 cells, cytoplasmic ROS were labeled with DCFH-DA and observed under a fluorescence microscope. The results showed that ROS was obviously activated after LPS+ATP stimulation, while PF treatment weakened the fluorescence intensity of ROS (Figure 1G,H). These results prove the cytoprotective effect of PF in vitro.

### 2.3. PF Protected BV2 Cells Against LPS+ATP-Induced NLRP3 Inflammasome Activation via SIRT1 Signaling In Vitro

**Figure 2:**
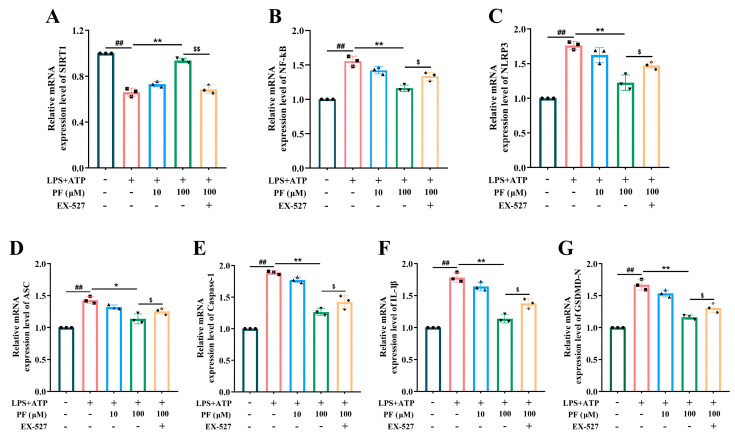
Effects of PF on protein levels of SIRT1, NF-κB and NLRP3 inflammasome in LPS+ATP treated BV2 cells. The relative mRNA expression level of (**A**) SIRT1, (**B**) NF-κB, (**C**) NLRP3, (**D**) ASC, (**E**) Caspase-1, (**F**) IL-1β, (**G**) GSDMD-N. Data are presented as mean ± SEM. ## *p* < 0.01, compared to control group; * *p* < 0.05, ** *p* < 0.01, compared to LPS+ATP group, $ *p* < 0.05, $$ *p* < 0.01 compared to PF+EX-527 group.

In order to reveal the potential mechanism related to the protective effect of PF on BV2 cells treated with LPS+ATP, we measured the expression levels of SIRT1 signal NF-κB and NLRP3 inflammasome by PCR. It was found that the expression of SIRT1 decreased, and the level of nuclear NF-κB p65 increased in BV2 cells stimulated by LPS+ATP (Figure 2A,B). At the same time, the expressions of NLRP3, Caspase-1 and GSDMD-N were up-regulated after LPS+ATP treatment (Figure 2C–G), whereas these changes were reversed by PF-100 μM. However, the treatment of SIRT1 inhibitor EX-527 significantly changed the protective effect of PF on BV2 cells treated with LPS+ATP and reversed the regulatory effect of PF on SIRT1 signal NF-κB and NLRP3 inflammasome. These findings reflect that SIRT1 signaling regulates inflammasome inactivation of NLRP3 in BV2 cells stimulated by LPS+ATP, which is related to PF-related protective efficacy.

### 2.4. PF Improved CUMS-Induced Depression-like Behavior in Mice

**Figure 3:**
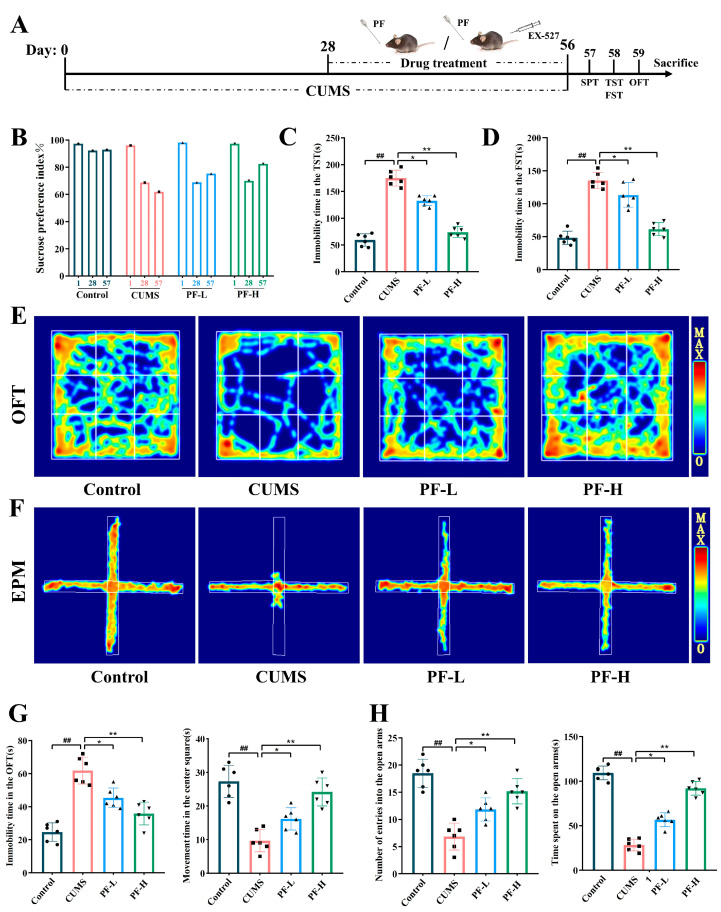
PF improved depressive-like behaviors in CUMS mice. (**A**) Experimental procedures. (**B**) SPT, (**C**) TST, (**D**) FST, (**E**,**G**) OFT and (**F**,**H**) EPM. Data are presented as mean ± SEM. ## *p* < 0.01, compared to control group; * *p* < 0.05, ** *p* < 0.01, compared to CUMS group.

The effect of PF on depression-like behavior in CUMS mice was evaluated by behavioral tests on mice. It was observed that the sucrose preference rate of mice in CUMS group was significantly lower than that in the control group, reflecting the state of pleasure deficiency in stressed mice (Figure 3B). Furthermore, compared with the control group, CUMS mice showed significantly longer immobility time in TST and FST, indicating that the stressed mice were desperate (Figure 3C,D). Compared with CUMS group, PF treatment significantly reversed sucrose preference and immobility time in FST. Additionally, the results of OFT showed that the immobility time of CUMS mice in OFT was significantly longer, and the exercise time of mice in the central area was significantly shortened, showing obvious depression-like behavior. However, after PF administration, the resting time in OFT and the time of crossing the central area of mice were obviously reversed (Figure 3E,G). The EPM investigates the anxiety state of animals by using the characteristics of animals’ exploration of new and exotic environments and their fear of hanging open arms. The EPM results found that the time and times of CUMS-induced mice entering the open arm were significantly reduced compared with the control group, and this phenomenon was significantly improved in PF-L and PF-H groups (Figure 3F,H).

### 2.5. PF Alleviated Neuroinflammation and Neuronal Damage in CA3 Area in Hippocampus of CUMS Mice

**Figure 4:**
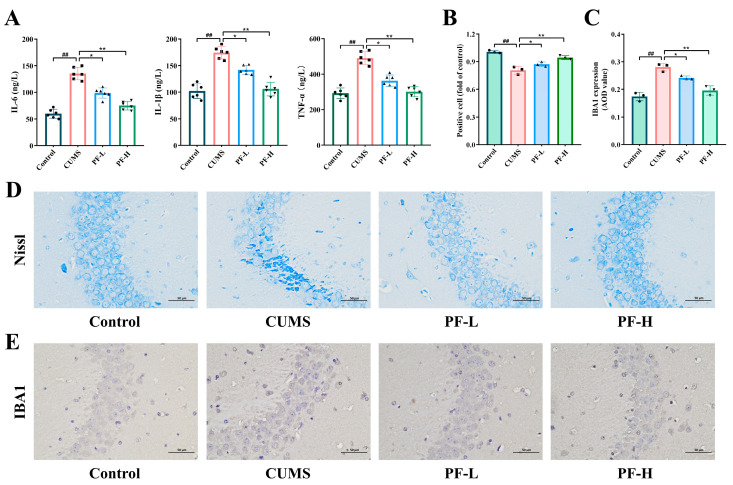
PF alleviated neuroinflammation and neuronal damage in CUMS mice. (**A**) The levels of IL-6, IL-1β, TNF-α. (**B**) Quantitative analysis of Nissl bodies positive cells. (**C**) The positive signal intensity of IBA1. (**D**) Nissl staining image (×200). (**E**) Immunohistochemical image of IBA1 (×200). Data are presented as mean ± SEM. ## *p* < 0.01, compared to control group; * *p* < 0.05, ** *p* < 0.01, compared to CUMS group.

In order to explore the effect of CUMS on hippocampal neuroinflammation in mice, we detected the expression level of inflammatory cytokines in the hippocampus of mice. The results showed that the expression levels of IL-6, IL-1β and TNF-α in the hippocampus of mice treated with CUMS were significantly higher than those of the control group, while the contents of IL-6, IL-1β and TNF-α in the hippocampus of mice were significantly decreased after different doses of PF treatment (Figure 4A). Then, Nissl staining was used to observe the changes of neurons in the CA3 area of mice induced by CUMS. The results suggested that CUMS stimulation led to dark staining of positive cells, disordered arrangement of nuclei and unclear nuclei compared with the control group. However, in the PF treatment group, nuclei and Nissl bodies with clear edges and orderly arrangement could be seen. These results indicate that PF administration can alleviate the neuroinflammation and neuronal damage in the CA3 area of CUMS mice (Figure 4B,D).

As a marker of microglia activation, IBA1 was up-regulated upon microglia activation, and the activation status of microglia was determined by immunohistochemical staining. The results showed that the number of IBA1-positive cells was increased in mice in the CUMS group compared with the control group. Additionally, PF-H significantly reduced the number of IBA1-positive cells, indicating that PF inhibited the activation of microglia in CUMS mice (Figure 4C,E).

### 2.6. Effect of PF on SIRT1 and NF-κB Expression in Hippocampus of CUMS Mice

**Figure 5:**
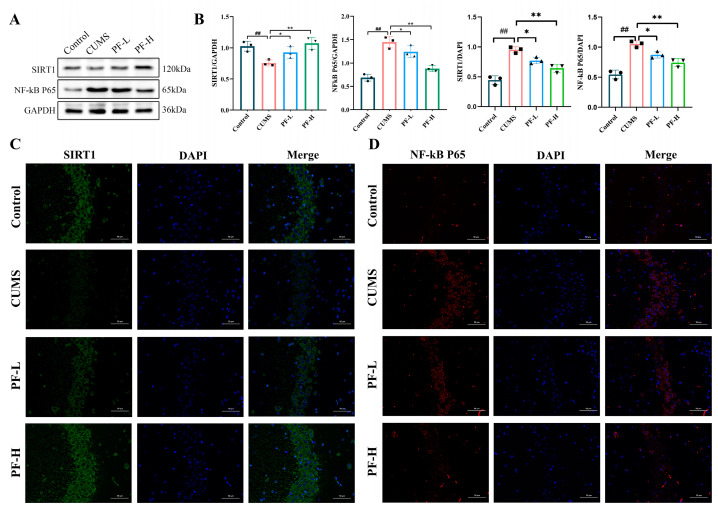
PF inhibited the activation of NF-κB by activating the expression of SIRT1. (**A**) Representative Western blots. (**B**) Quantification of SIRT1 and NF-κB P65 expression levels and fluorescence intensity. (**C**) The fluorescence intensity of SIRT1 (×400). (**D**) The fluorescence intensity of NF-κB P65 in CA3 area of hippocampus (×400). Data are presented as mean ± SEM. ## *p* < 0.01, compared to control group; * *p* < 0.05, ** *p* < 0.01, compared to CUMS group.

In order to evaluate the effect of PF on the expression of SIRT1 and NF-κB P65 in CUMS mice, we performed immunoblotting and immunofluorescence analysis. The results showed that CUMS treatment reduced the expression level of SIRT1 in the hippocampus of mice slightly, compared with the control group (Figure 5A,B) and significantly reduced the fluorescence intensity (Figure 5B,C), while PF-H significantly restored the fluorescence intensity and expression level of SIRT1. In contrast, the protein expression and fluorescence intensity of NF-κB P65 were significantly enhanced after CUMS induction compared with the control group, while PF-H administration significantly inhibited the expression level of NF-kB P65 (Figure 5A,B,D) and weakened the fluorescence intensity of NF-κB P65 (Figure 5B,C). It is suggested that PF activated the expression of SIRT1 in mouse hippocampus induced by CUMS and inhibit the activation of NF-κB P65.

### 2.7. Effects of PF on NLRP3 Inflammasome and Pyroptosis in the Hippocampus of CUMS Mice

**Figure 6:**
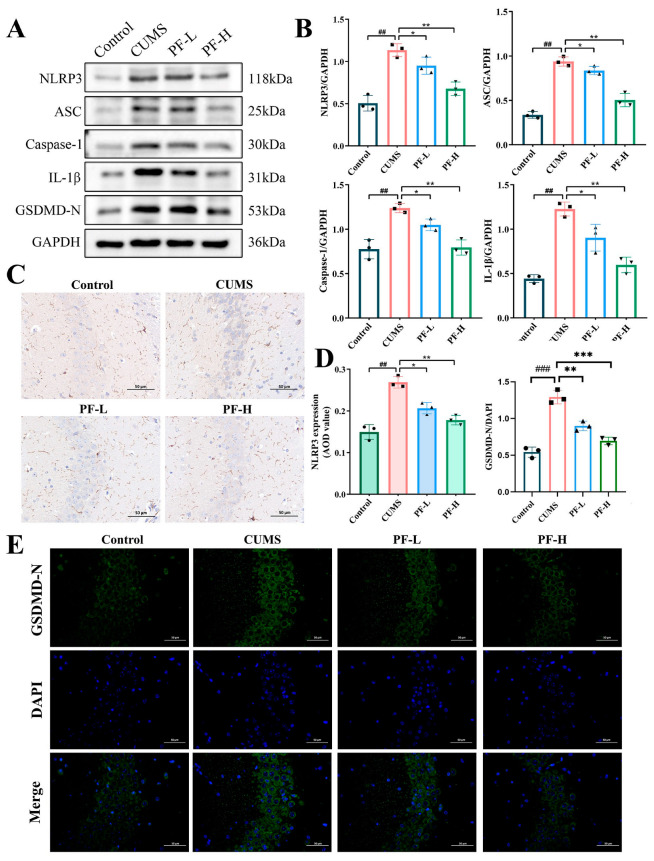
PF inhibited the activation of NLRP3 inflammasome and its mediated pyroptosis. (**A**) Representative Western blots. (**B**) The quantitative analysis of the expression level of NLRP3, ASC, Caspase-1, IL-1β and GSDMD-N. (**C**) The positive signal intensity of NLRP3 (×200). (**D**) IHC quantitative analysis of NLRP3. (**E**) The fluorescence intensity of GSDMD-N/DAPI in CA3 area of hippocampus (×400). Data are presented as mean ± SEM. ## *p* < 0.01, ### *p* < 0.001, compared to control group; * *p* < 0.05, ** *p* < 0.01, *** *p* < 0.001 compared to CUMS group.

Western blot was used to evaluate the effect of PF on the activation of inflammasome in hippocampus NLRP3. The results showed that the protein expression levels of NLRP3, ASC and Caspase-1 related to the assembly of NLRP3 inflammasome in the hippocampus of mice (exposed to CUMS) were significantly increased. Moreover, the activation of the NLRP3 inflammasome mediated the release of inflammatory cytokines and the activation of pyroptosis in the hippocampus of CUMS mice, which showed that the expression of IL-1β and GSDMD-N in CUMS group increased (Figure 6A,B). Nevertheless, PF administration decreased the expression level of these proteins to different degrees, and PF-H significantly inhibited the activation of inflammatory corpuscles and pyroptosis of NLRP3 inflammasome.

Furthermore, the results of IHC suggested that the positive signal intensity of NLRP3 in the CA3 region was enhanced after 28 days of CUMS, compared with the control group, and PF-H treatment could reverse this change (Figure 6C,D). Further immunofluorescence results indicated that the relative fluorescence intensity of GSDMD-N in the CA3 region of the model group increased significantly. In contrast, PF-H administration significantly reduced the relative fluorescence intensity of GSDMD-N/DAPI in CUMS mice (Figure 6D,E).

### 2.8. Effect of SIRT1 Blockage on PF-Related Improvements of NF-κB, NLRP3 Inflammasome and Pyroptosis

**Figure 7:**
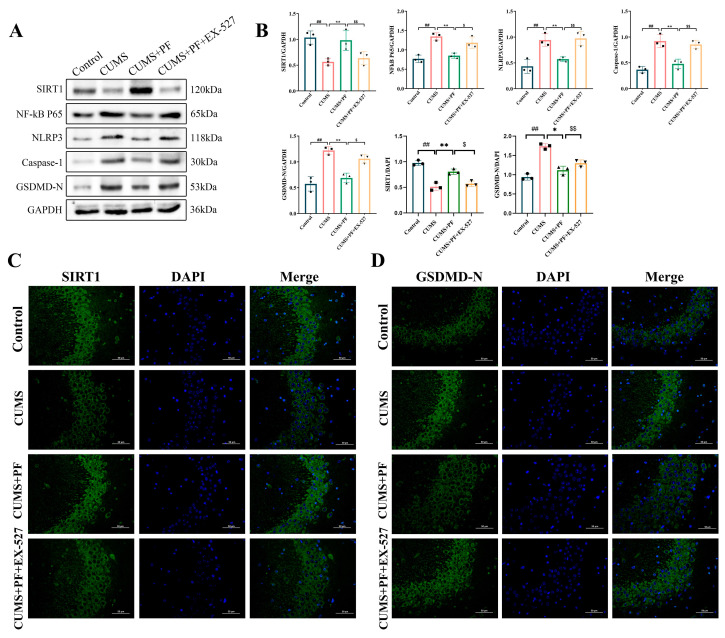
EX-527 blocked the inhibitory effect of PF on the activation of NF-κB, NLRP3 inflammasome and pyroptosis in CUMS model mice. (**A**) Representative Western blots. (**B**) Quantification of SIRT1, NF-kB-P65, NLRP3, Caspase-1 and GSDMD-N expression levels and quantification of SIRT1 and GSDMD-N fluorescence intensity. (**C**) The fluorescence intensity of SIRT1/DAPI in CA3 area of hippocampus (×400). (**D**) The fluorescence intensity of GSDMD-N/DAPI in CA3 area of hippocampus (×400). Data are presented as mean ± SEM. ## *p* < 0.01, compared to control group; * *p* < 0.05, ** *p* < 0.01, compared to CUMS group, $ *p* < 0.05, $$ *p* < 0.01 compared to PF+EX-527 group.

In order to further explore the role of SIRT1 signaling in PF-related antidepressants, EX-527, a SIRT1-specific inhibitor, was used in this study. The results indicate that the activation of SIRT1 expression by PF in the hippocampus of CUMS mice is obviously reversed after EX-527 treatment (Figure 7A,B). Simultaneously, the immunofluorescence results show that the relative fluorescence intensity of SIRT1 in the CA3 area of the hippocampus in CUMS group is obviously weakened compared with that in the control group, and the fluorescence intensity is obviously enhanced in the PF group, but this effect of PF is significantly reversed after EX-527 treatment (Figure 7B,C).

Then, we verified whether SIRT1 signaling also regulated the inhibitory effect of PF on the activation of NLRP3 inflammasome. As a result, the SIRT1 antagonist EX-527 reversed the down-regulation of PF on the expression levels of NF-κB p65, NLRP3, Caspase-1 and GSDMD-N (Figure 6A,B). Similarly, the immunofluorescence image of GSDMD-N showed that the inhibitory effect of PF on the expression of pyroptosis-related protein GSDMD-N in the CA3 area of mouse hippocampus induced by CUMS was obviously weakened by the treatment of EX-527 (Figure 7B,D). These results indicate that the inhibitory effect of PF-related inactivation of NLRP3 inflammasome and pyroptosis depended on SIRT1 signal transduction (Figure 7A,B).

## 3. Discussion

In this study, we used the LPS+ATP-induced microglia model and CUMS-induced mice model to explore the antidepressant characteristics of paeoniflorin and its possible mechanism. Our data found that after paeoniflorin treatment, the depression-like behavior of mice is obviously weakened, the neuronal damage is alleviated, and the neuroinflammation is reduced. In addition, the protein expression of key regulatory factors in the SIRT1/NF-κB/NLRP3/GSDMD pathway can also be changed with the treatment of paeoniflorin. In order to further explore the antidepressant mechanism of paeoniflorin, SIRT1 inhibitor EX-527 was used in animal experiment 2 and BV2 cells. Interestingly, we found that the therapeutic effect of paeoniflorin was changed by blocking the expression of SIRT1, which indicated that SIRT1 and NLRP3/GSDMD played a key role in the relief of depression by paeoniflorin.

It is found that glial cells are important cellular components in the central nervous system except for neurons [23]. Among glial cells, microglia, as a type of innate immune cell in the central nervous system, can assist the formation of synapses in the central nervous system, maintain brain homeostasis, and play an important role in host defense and tissue repair [24]. However, when microglia are improperly or continuously activated, the activated microglia will secrete various inflammatory cytokines to the extracellular environment [25] and participate in the generation of reactive oxygen species (ROS), which will eventually destroy the surrounding neurons and produce neurotoxicity to the surrounding neurons [26]. Therefore, it is necessary to further understand the detailed mechanism of microglia activation, especially the interaction between inflammation and ROS, in order to determine potentially more effective therapeutic targets. In our study, the fluorescence results of ROS showed that BV2 cells were activated after LPS and ATP induction. Overactivation of microglia can induce pyroptosis, as shown by LDH and PI measurements in vitro. Pyroptosis induced by LPS and ATP is characterized by cell membrane permeability and apoptosis. In addition, the secretion of Caspase-1, IL-1β and IL-18 increased. These findings support the existence of burnt cells, and PF can reverse this death, indicating that PF has a certain protective effect on BV2 cells.

CUMS is a classic model of depression. In real life, people often encounter different, unpredictable mild stress, which leads to chronic and long-term stress. It is found that in this model, long-term exposure of rodents to certain stimuli or pressures will lead to an obvious lack of pleasure, behavioral despair and decreased responsiveness. Similar to the previous research results, mice stimulated by CUMS showed a decrease in sucrose preference rate, an increase in the resting time of TST and FST, a decrease in the time and times of OFT passing through the central area, and an increase in the time and distance of open-arm movement in EPM, suggesting that depression occurred after CUMS stimulation. Paeoniflorin can significantly improve the behavior defects of SPT, TST, FST and OFT in mice, suggesting that paeoniflorin can effectively alleviate the depression-like behavior in mice.

Neuroinflammation is an essential phenomenon in the pathogenesis of mental illness, which is related to the pathogenesis of depression. More and more studies indicated that inflammation mediated by activated microglia plays an important role in the pathogenesis of many nervous systems and neurodegenerative diseases, such as cerebral ischemia, Alzheimer’s disease, depression and Parkinson’s disease [27]. In this study, mice exposed to CUMS showed elevated levels of IL-6, IL-1β and TNF-α in serum. Here, we found that IBA1 staining showed that microglia in the hippocampus of mice were activated after CUMS induction. The results showed that CUMS induced inflammatory reaction and activation of microglia in the hippocampus of mice. In addition, Nissl staining showed that CUMS damaged neurons in the CA3 area of the hippocampus of mice, which affected the morphology and quantity of Nissl bodies in the hippocampus. These phenomena were obviously improved after PF treatment, which indicated that PF could improve nerve inflammation and neuron injury induced by CUMS.

NLRP3 inflammasome is an important part of the immune system, and it is composed of NLRP3, ASC and Caspase-1 [28]. The activation of the NLRP3 inflammasome complex promotes the activation of Caspase-1 and the maturation of IL-1β and IL-18 into active cytokines, which further induces cell division [29]. Additionally, the terminal structure of GSDMD-N has pore-forming activity, which eventually leads to cell rupture and further induces pyroptosis [30]. NLRP3 inflammasome exists in microglia and astrocytes of the central nervous system [31]. Our experimental results showed that the mRNA expressions of NLRP3, ASC and Caspase-1 in BV2 cells induced by LPS+ATP are up-regulated, and the protein levels of NLRP3 and ASC in animal hippocampus treated by CUMS are also increased. However, NLRP3 and ASC stimulate the cleavage of Caspase-1, which will lead to the cleavage of gasdermin-D and IL-1β. As in previous studies, our experimental results show that IL-1β and GSDMD-N are activated by LPS+ATP and CUMS. These results indicate that inflammasome activation of NLRP3 is closely related to depression-like symptoms and microglia activation in mice. The promotion of NLRP3 inflammatory corpuscles will activate GSDMD-N, which will lead to pyroptosis, indicating that inhibiting pyroptosis is an effective treatment strategy to prevent depression. The experimental results of this study displayed that PF can alleviate GSDMD-mediated pyroptosis to some extent by inhibiting neuroinflammation and activation of NLRP3 inflammasome. Here, we proved the inhibitory effect of PF on GSDMD-mediated microglia pyroptosis.

SIRT1/NF-κB signaling has been proven to regulate the inflammasome of NLRP3 and is related to various diseases [32]. Existing evidence shows that SIRT1 plays an anti-inflammatory role by interfering with the NF-kB signal [33]. NF-κB is a transcription factor that plays an important role in the development of many inflammatory reactions [34]. Once NF-κB is activated, NF-κB will translocate into the nucleus and increase the signal activation of IL-1β and NLRP3 [35]. In addition, pyroptosis mediated by NLRP3 can be regulated by NF-κB, which participates in various inflammatory reactions through the transcription of pro-inflammatory factors [36]. In our study, compared with the model group, with the treatment of PF, the expression level of SIRT1 increased significantly, and the expressions of NF-kB, NLRP3, ASC, Caspase-1, IL-1β and GSDMD-N decreased significantly. Moreover, SIRT1 inhibitor EX-527 hindered the protective effect of paeoniflorin in LPS+ATP-treated cells and CUMS-induced mice and promoted the expression of NF-κB, NLRP3, ASC, Caspase-1, IL-1β and GSDMD-N. In this study, we found that the activation of SIRT1/NF-κB induced by PF significantly reduced the activation of NLRP3 inflammasome complex and its induced pyroptosis in CUMS mice and BV2 cells, while SIRT1 inhibitors blocked these effects. It is confirmed that paeoniflorin can regulate inflammatory corpuscles and pyroptosis of NLRP3 through SIRT1/NF-κB signaling, and SIRT1 signaling plays an important role in antidepressant action.

## 4. Materials and Methods

### 4.1. Chemicals and Reagents

Paeoniflorin, LPS, ATP (Yuanye, Shanghai, China); Selisistat (EX-527) (MCE, Shanghai, China); DMEM culture medium (Gibco, Shanghai, China); premium fetal bovine serum (FBS) (Clarke Bioscience, Webster, TX, USA); PBS buffer solution, TBST solution, tris-glycine SDS-PAGE electrophoresis buffer, transmembrane buffer (Solarbio, Beijing, China); polyvinylidene difluoride (PVDF) membranes (Merck Millipore, Darmstadt, Germany); TrypsinSolution (EDTA-free), penicillin-streptomycin solution, 4% paraformaldehyde (PFA), skim milk powder, color prestained protein marker (Biosharp, Hefei, China); loading buffer (Beyotime, Haimen, China); anti-NLRP3 rabbit, anti-ASC rabbit, anti-Caspase-1 rabbit, anti-LC3 rabbit (Proteintech, Wuhan, China), anti-IL-1β rabbit, anti-SIRT1 rabbit, anti-NF-κB rabbit (Wanleibio, Shenyang, China), anti-GAPDH rabbit, anti-GSDMD-N rabbit, HRP-labeled goat anti-rabbit IgG, Alexa Fluor 488 labeled goat anti-rabbit IgG, DAPI staining reagent (Servicebio, Wuhan, China); IL-1β ELISA kit: Human IL-1β ELISA kit (ELISA) Beyotime Biotechnology Co. Ltd. (Shanghai, China); IL-18 ELISA kit: Human IL-18 ELISA kit (ELISA) Beyotime Biotechnology Co. Ltd. (Shanghai, China) ; IL-6 ELISA kit: Human IL-6 ELISA kit (ELISA) Beyotime Biotechnology Co. Ltd. (Shanghai, China); TNF-α ELISA kit: Human TNF-α ELISA kit (ELISA) Beyotime Biotechnology Co. Ltd. (Shanghai, China).

### 4.2. Cells Cultures and Treatments and Cell Viability CCK-8 Assay

Microglia BV2 line (purchased from Shanghai CAS Cell Bank, Shanghai, China) was cultured in 10% FBS and 1% penicillin/streptomycin DMEM medium at 37 °C with 5% CO_2_ concentration. BV2 cells in the logarithmic growth phase were inoculated into 96-well plates, 10^4^ cells/well. The BV2 cells were treated with LPS (1 µg/mL) for 4 h and ATP (5 mM) for 1 h, respectively. Then, the administration group was given 100 μL of paeoniflorin with different concentrations (1, 10, 50, 100, 200, 400 μM) for 4 h.

The absorbance of cells in each group at 450 nm was detected by cell counting kit 8 (CCK-8 Kit, Beyotime, Shanghai, China) in an enzyme-labeled instrument to evaluate the cell viability.

### 4.3. Determination of Caspase-1 and LDH Expression Levels in Cells

Pyroptosis is a programmed pyroptosis dependent on Caspase-1. Therefore, we used Caspase-1 kits to detect the concentration of Caspase-1 in BV2 cells. Pyroptosis will lead to the release of lactate dehydrogenase (LDH) from the cell to the outside of the cell. We detected the concentration of LDH in the cell culture solution by using an LDH kit. The purpose is to indirectly reflect the degree of BV2 pyroptosis through the above indicators.

### 4.4. Determination of the Expression Levels of IL-1β and IL-18 in Cells

The expression levels of IL-1β and IL-18 in the supernatant of BV2 cells were determined by IL-1β ELISA kit and IL-18 ELISA kit. Firstly, 50 μL different concentrations of standards were added into each standard well. Then, 10 μL of the samples were added into the sample wells, and 40 sample diluent was added. Secondly, 100 μL horseradish peroxidase (HRP)-labeled detector antibody was added into each well and incubated for 60 min at 37 °C. Then, 50 μL substrate A and 50 μL substrate B were added into each well and incubated at 37 °C for 15 min without light. Finally, 50 µL termination fluid is added to each well. Subsequently, the OD value of each well was measured at 450 nm after 15 min.

### 4.5. Hoechst/PI Double Staining

In order to quantify apoptotic cells, BV2 cells were incubated for 20 min by adding 5 μL Hoechst 33,258 stain and 5 μL PI stain. The morphological characteristics of apoptosis (high-density fluorescence with nuclear contraction, chromatin fragmentation and concentration) were observed and monitored by fluorescence microscope.

### 4.6. Intracellular and Mitochondrial ROS Detection

BV2 cells were routinely cultured at a density of 5 × 10^4^/mL in a logarithmic growth period and inoculated in 6-well plates for 24 h. 20 min before the end of drug treatment, DCFH-DA was added to each well at the ratio of 1:1000 so that the probe was in complete contact with the cells. After incubation at 37 °C for 20 min, PBS was washed three times. The fluorescence intensity of ROS was observed under a fluorescence microscope.

### 4.7. CUMS Procedure and Experimental Design

Male 6–8 week-old C57BL/6 mice weighing 20–22 g were purchased from Changchun Yisi Experimental Animal Center. The experimental animals were raised under standard feeding conditions, with 12 h light/12 h dark cycle at 18–22 °C, and were allowed to eat and drink freely during the experiment. All experiments were conducted with permission from the Jilin Agriculture University Ethics Committee of Experimental Animals (No. 2024 08 27 001).

The procedure of CUMS is as follows [37]. During the whole experiment, some stress factors were applied separately and continuously, including deprivation of food or water, foreign bodies in the cage, soaking of the rat cage, oscillating squirrel cage, thermal stress, cage tilting at 45 degrees, upside down day and night, space shrinking and predator’s voice. The animals in the control group were placed in a separate environment and had no contact with the animals in the model group. In order to ensure the unpredictability of stressors, all stressors were randomly applied. The whole CUMS was carried out within 56 days.

#### 4.7.1. Experiment 1

After a week of adaptive feeding, mice were randomly divided into 4 groups (*n* = 8): control group (control), model group (CUMS), low-dose paeoniflorin group (PF-L) and high-dose paeoniflorin group (PF-H). Except for the control group, all groups were given CUMS modeling. From the 28th day, the control and CUMS groups were given distilled water (20 mL/kg/d), while the PF-L and PF-H groups were given paeoniflorin (40, 80 mg/kg/d, respectively). The weight of each group was measured every week, and the behavior test was carried out on the 28th and 56th day.

#### 4.7.2. Experiment 2

After a week of adaptive feeding, mice were randomly divided into 4 groups (*n* = 8): control group (Control), model group (CUMS), paeoniflorin group (PF) and paeoniflorin + SIRT1 inhibitor-EX-527 group (PF+EX-527). Except for the control group, all groups were given CUMS modeling. From the 28th day, the control and CUMS group were given distilled water (20 mL/kg/d), and paeoniflorin (80 mg/kg/d) was given to the PF group and PF+EX-527 group by gavage. At the same time, EX-527 (10 mg/kg/d) [38] was injected intraperitoneally into the PF+EX-527 group.

Finally, eyeball blood from all mice was taken and then killed. The hippocampus of mice was fixed in 4% paraformaldehyde or frozen at −80 °C until the next experiment. The experimental design procedure is shown in Figure 3A.

### 4.8. Behavioral Experiment

#### 4.8.1. Sucrose Preference Test (SPT)

Before the experiment, the sucrose training was conducted to confirm the normal sucrose preference of animals. During the experiment, the mice were kept in a separate cage and were free to use two bottles of sucrose solution and water. Put two bottles of 1% sucrose solution in each cage for 24 h, and then replace one bottle of sucrose solution with one bottle of water for 24 h. After adaptation, mice were deprived of water for 12 h. After 12 h, the consumption of sucrose solution and water were recorded respectively. Sucrose preference was calculated according to the following equation:

sucrose preference = (sucrose consumption)/(total fluid consumption) × 100%

#### 4.8.2. Tail Suspension Test (TST)

Mice were tied approximately 1 cm from the tail tip and suspended approximately 20 cm from the floor. The test was performed in a dark, quiet room. The experiment lasted 6 min and was recorded manually with a video camera. Total resting time was obtained by observing the last 4 min of resting time of the mice in the video. To avoid randomness, each experiment should be repeated three times. This state of immobility is defined as the mouse having stopped struggling or not having any activity.

#### 4.8.3. Forced Swimming Test (FST)

Mice were placed in an open cylindrical Plexiglas container (20 cm high, 14 cm in diameter) with 10 cm deep water (25 ± 1 °C). The mice are forced to swim for 6 min with a final rest period of 4 min, which is recorded manually with a video camera, and the total rest period is obtained by observing the last 4 min of the rest of the mice in the video. Each experiment should be repeated three times to avoid randomization. This state of immobility is defined as the mouse having stopped struggling or not having any activity.

#### 4.8.4. Open Field Test (OFT)

OFT was used to assess anxiety and locomotor activity in rodents. Mice were gently placed in an open room (80 × 80 cm) equipped with a camera. During the test, the mice were allowed to explore the space for 5 min. Corresponding time data were acquired via the built-in camera and analysis software of the test device. The total distance moved by the mice, the number of passes through the center area, and the time spent in the center area are automatically recorded. Each experiment should be repeated three times to avoid randomness.

#### 4.8.5. Elevated Plus Maze (EPM)

The EPM device consists of an open arm and a closed arm. Before the experiment, the designated activity area was set, and the mice were placed in the center area of the maze and allowed to move freely in the maze for 5 min. Relevant time data were obtained through the built-in camera and analysis software of the test device. The number of times the mice entered the open arm, the time they moved in the open arm and the trajectory were recorded by the camera. Each experiment should be repeated three times to avoid randomness.

### 4.9. Determination of IL-1β, IL-6 and TNF-α in Serum

After behavioral testing, the serum was collected. The expression levels of IL-1β, IL-6 and TNF-α in serum were detected using IL-1β, IL-6 and TNF-α ELISA kits. First, 50 μL different concentrations of standards were added into each well. Then, 10 μL of samples were added into the sample wells, and 40 μL sample diluent was added. Second, the 100 μL horseradish peroxidase (HRP)-labeled detection antibody was added into each well and incubated at 37 °C for 60 min. After that, 50 μL substrate A and 50 μL substrate B were added into each well and incubated at 37 °C for 15 min without light. Finally, 50 μL termination solution is added. In addition, the OD value of each well was measured at 450 nm after 15 min.

### 4.10. Nissl Staining

The mice’s brain tissue was fixed by PFA, embedded in paraffin, sliced, dehydrated in ethanol, soaked in xylene and rehydrated in ethanol. Hydrate with 1% toluidine blue at 60 °C for 30 min, then dehydrate with ethanol to remove xylene, and finally seal the film. The morphology and number of neurons in the hippocampus were observed under an optical microscope.

### 4.11. Immunofluorescence

Set the temperature of the cryosectioner to −20 °C. A layer of OCT embedding gel was applied to the black chassis of the section beforehand and then freeze-fixed for 2 min. After removal, the surface was flattened with a razor blade. The mouse brain sections were covered with OCT embedding gel. Then, the brain slices were cut into 30 μm thickness. Mouse brain slices were washed with PBS for 5 min × 3 times and sealed in PBS containing 10% goat serum and 0.3% Triton X-100 for 1 h at room temperature. The slices were incubated with anti-SIRT1, anti-NF-κB, and anti-GSDMD at 4 °C overnight. The next day, the slices were incubated with Alexa Fluor Plus 488 at room temperature for 1 h. The slices were washed with PBS for 3 times (5 min each time). Images were obtained using an inverted fluorescence microscope at 594 nm and analyzed using ImageJ software v5.2.

### 4.12. Immunohistochemistry

Using the same method as IF staining to make sections and antigen retrieval, then inactivating endogenous peroxidase activity and PBS washing. After being blocked with 5% goat serum for 15 min, the slices were incubated with anti-IBA1 or anti-NLRP3 overnight at 4 °C, and the slices were incubated with goat anti-rabbit IgG polymer for 30 min. Finally, 3,3-diaminobenzidine was used to observe the immune response, and then hematoxylin was retained. The image was taken by an optical microscope.

### 4.13. Western Blot

RIPA lysis buffer containing 1% PMSF solution was added to the hippocampus, then ground and lysed in an ice bath for 30 min, and then centrifuged at 14,000 rpm for 15 min, and the supernatant was extracted. The protein concentration was determined by the BCA protein analysis kit. Mix the albumin sample with 5× loading buffer 1:4, boil it for 5 min, and store it in the refrigerator at −80 °C. The samples were separated by 12% SDS-PAGE and transferred to the PVDF membrane. The membrane was sealed with 5% skim milk at 37 °C for 2 h and then incubated with the primary antibody at 4 °C overnight. The membrane was incubated with the HRP-labeled goat anti-rabbit IgG (H + L) for 2 h at room temperature. The membrane was treated with an ELC chemiluminescence solution, imaged under a Chemiluminescence Imager (Analytik Jena, Jena, Germany), and analyzed with ImageJ software v5.2.

### 4.14. Reverse Transcription (RT)-and Real-Time Quantitative Polymerase Chain Reaction (qPCR)

Total RNA was isolated from BV2 cells after drug treatment with a Trizol reagent and quantified with a NanoDrop 2000 spectrophotometer (Thermoscientific, Bremen, Germany). According to the manufacturer’s instructions, a primescript RT reagent kit was used for reverse transcription. Real-time qPCR was performed on a StepOnePlus instrument (Analytik Jena, Jena, Germany) using the SYBR Green Master Mix (Servicebio). The steps of the amplification procedure are 95 °C 30 s, 95 °C 3 s 40 cycles and 60 °C 30 s. The relative mRNA expression was calculated by the 2^−∆CT^ method, and GAPDH was used as endogenous control. Primers were synthesized by Takara Biomedical Technology, and the primer sequences are shown in Table 1.

### 4.15. Statistical Analysis

All statistical analyses were conducted using GraphPad Prism 8. Results were statistically evaluated using one-way analysis of variance (ANOVA). Data are presented as mean ± standard error of the mean. *p* < 0.05 were considered significant.

## 5. Conclusions

This study investigated the antidepressant characteristics of PF and its potential mechanism. We found that PF has a protective effect on BV2 cells treated with LPS+ATP in vitro and can effectively alleviate the depression-like behavior of CUMS mice in vivo. This effect is related to SIRT1/NF-κB signal transduction stimulating the inactivation of NLRP3 inflammasome. PF inhibits the production of pro-inflammatory cytokine IL-1β and pyroptosis executor GSDMD-N, thus inhibiting the inflammatory activation of microglia and exerting neuroprotective effects in vivo and in vitro. However, the in vivo and in vitro effects related to PF are weakened by the blocking of SIRT1 signal transduction. In a word, this study indicates that PF can effectively alleviate the depression-like behavior of CUMS mice, which depends on the SIRT1 signal to regulate the activation of NLRP3 inflammasome and pyroptosis.

## Figures and Tables

**Table 1:** The table of primer sequence information.

	Forward	Reverse
Gapdh	5′-TGTTTCCTCGTCCCGTAGA-3′	5′-GATGGCAACAATCTCCACTTTG-3′
Sirt1	5′-TGGACGAGCTGACCCTTGA-3′	5′-TCCTGCGGATGTGGAGATT-3′
Nf-kb	5′-AGAGCAACCGAAACAGGAGG-3′	5′-TTTGCAGGCCCCACATAGTT-3′
Nlrp3	5′-CTCGCATTGGTTCTGAGCTC-3′	5′-AGTAAGGCCGGAATTCACCA-3′
Asc	5′-TTGCTGGATGCTCTGTATGG-3′	5′-CCAAGTAGGGCTGTGTTTGC-3′
Caspase-1	5′-CTGACTGGGACCCTCAAGTT-3′	5′-TCAACTTGAGCTCCAACCCT-3′
Il-1β	5′-GTTCCCATTAGACAACTGC-3′	5′-GATTCTTTCCTTTGAGGC-3′
Gsdmd-n	5′-AAGATCGTGGATCATGCCGT-3′	5′-AACGGGGTTTCCAGAACCAT-3′

## Data Availability

The original contributions presented in the study are included in the article, further inquiries can be directed to the author.
